# Supplementary material for: Collaborative intelligence in AI: Evaluating the performance of a council of AIs on the USMLE
Source: PLOS Digit Health. 2025 Oct 9;4(10):e0000787. doi: 10.1371/journal.pdig.0000787 (PMC12510544; doi:10.1371/journal.pdig.0000787)
Supplement: S2 File — (ZIP) [file pdig.0000787.s002.zip › S2 Data Raw data files containing unprocessed question inputs and LLM outputs including transcripts of deliberations/STEP2_Question-99-1_Type-MC-NJ.docx]

**Facilitator** **[Round 1]:** A clinical trial is performed to assess the efficacy of aspirin in decreasing the risk for myocardial infarction. Twenty-two thousand male physicians with no history of coronary artery disease are randomly assigned to receive aspirin (325 mg/d) or a placebo. After following the subjects for 5 years, results show that the incidence of myocardial infarction is 1.2% in the aspirin group and 2.2% in the placebo group. The reduction in incidence is statistically significant. Which of the following is the most accurate interpretation of these results? (A) Absolute risk reduction is 1% (B) Absolute risk reduction is 10% (C) Number needed to treat is 4 (D) Number needed to treat is 25 (E) Relative risk reduction is 25% (F) Relative risk reduction is 75%

**AI_D:** RESTATING THE QUESTION: This question asks for the most accurate interpretation of the trial results based on the given data. Essentially, the study sought to understand whether aspirin could effectively reduce the risk of myocardial infarction, which is the medical term for a heart attack. The incidence of heart attacks in two different groups, one receiving aspirin and the other placebo, is given for analysis. Different measures such as the Absolute Risk Reduction (ARR), Number Needed to Treat (NNT), and Relative Risk Reduction (RRR) are common statistical measures used to interpret the results of studies like this one. PLANNING: To find the most accurate interpretation, we must first calculate the ARR, NNT, and RRR. The ARR is simply the difference between the incidence in the placebo group and the incidence in the treatment group. NNT, on the other hand, is basically how many patients need to be treated with the drug for one patient to benefit. It is calculated by taking the reciprocal of the ARR. Lastly, the RRR is calculated by dividing the ARR by the incidence in the control group. CLINICAL REASONING: From the given data, the incidence of myocardial infarction in the aspirin group is 1.2% and in the placebo group it is 2.2%. This means that the ARR is 2.2% - 1.2% = 1.0%. In terms of proportions, this is 0.022 - 0.012 = 0.01. The NNT would then be 1 / 0.01 = 100. Therefore, one hundred patients need to be treated with aspirin for one patient to avoid a myocardial infarction. Lastly, the RRR is calculated as 0.01 / 0.022 = 0.45, or 45%. CONCLUDING: Observing the given choices, (A) Absolute risk reduction is 1% fits the calculated ARR value. The NNT calculated (100) does not match with the provided NNT values in choices (C) or (D). The RRR value calculated (45%) is not available in the given options (E) and (F). Thus, the most accurate interpretation of these results among the provided choices would be (A) Absolute risk reduction is 1%. RESPONSE: (A) Absolute risk reduction is 1%.

**AI_B:** 1) RESTATING THE QUESTION: The question is asking how to interpret the results of a clinical trial that examined the effect of aspirin on the risk of myocardial infarction, after comparing the incidences in an aspirin-treated group and a placebo group. Key terms like absolute risk reduction, relative risk reduction, and number needed to treat are statistical measures often used in biomedical research to measure the effectiveness of an intervention. 2) PLANNING: For the assumptions, we can see that the incidence of myocardial infarctions decreased from 2.2% to 1.2% in those taking aspirin. The assumptions we can generate from this include the possible reduction in absolute or relative risk, and the number of individuals who need to take the aspirin to prevent one case of myocardial infarction (Number Needed to Treat or NNT). In order to decide which assumption should be pursued further, we need to calculate based on the given figures. 3) CLINICAL REASONING: Let's examine each assumption clinically. (A) Absolute Risk Reduction (ARR) is the difference in events rates between the control group and the treatment group. In this case, it would be 2.2% (placebo group) - 1.2% (aspirin group) = 1% so this seems probable. (B) Absolute risk reduction cannot be 10% as the incidences do not provide this difference. (C) The Number Needed to Treat (NNT) is the inverse of the absolute risk reduction converted to a whole number (1/ARR). If the ARR is 1%, the NNT = 1/0.01 = 100. (D) Based on the calculations, the NNT cannot be 25. (E) Relative Risk Reduction (RRR) is the absolute risk reduction / control event rate (CER). Here it would be 1% / 2.2% = 0.455 or 45.5%. (F) From our calculations, the RRR cannot be 75%. 4) CONCLUDING: From a step-by-step examination of the data and our calculations, the most accurate interpretation of these results appears to be that the absolute risk reduction is 1%. 5) RESPONSE: Therefore, the correct answer is (A) Absolute risk reduction is 1%.

**AI_C:** This question asks for an accurate interpretation of the results of a clinical trial assessing the efficacy of aspirin in decreasing the risk for myocardial infarction among 22,000 male physicians without coronary artery disease. The results show that the incidence of myocardial infarction was 1.2% in the aspirin group and 2.2% in the placebo group, and the reduction in incidence is statistically significant. One possible assumption in this scenario is that the risk reduction is either absolute or relative, and the options provide various values for these. Another line of thought could come from considering the Number Needed to Treat (NNT), which quantifies the number of patients who need to be treated to prevent one additional bad outcome. From a clinical perspective, the difference in incidence rates between the placebo and aspirin group indicates the Absolute Risk Reduction (ARR), which is calculated by subtracting the event rate in the treated group from the event rate in the control group (2.2% - 1.2% = 1%). This seems to align with option A. Relative Risk Reduction (RRR) is calculated by dividing the ARR by the event rate in the control group (1.0%/2.2% = ~45%) which does not align with either of the given options E or F. Number Needed to Treat is 1/ARR, expressed as a percentage. Thus, NNT = 1/0.01 = 100, which also does not align with options C or D. Taking into consideration these calculations and comparisons, our analysis suggests that the most accurate interpretation of these results is that the Absolute Risk Reduction is 1% (Option A). That is, for every 100 individuals treated with aspirin, there is one less myocardial infarction compared to placebo over this 5-year period. This analysis points to the conclusion that the aspirin therapy can indeed reduce the risk of myocardial infarction, highlighting the importance of interventions in preventative health, especially in at-risk populations. However, it's crucial to remember that aspirin therapy also carries its own risks, such as gastrointestinal bleeding, and these factors should be considered in any decision to implement widespread preventative measures. So, the most accurate interpretation of these results is "Absolute risk reduction is 1%" (Option A).

**AI_A:** 1) RESTATING THE QUESTION: The query given concerns the interpretation of results from a clinical trial performed to check the efficacy of aspirin in reducing the risk of a myocardial infarction. The participants are 22,000 male physicians without any prior history of coronary artery disease. They were randomly assigned to receive either aspirin or a placebo. After five years, the incidence of myocardial infarction was 1.2% in the aspirin group and 2.2% in the placebo group. Various statistical measures, namely Absolute risk reduction, Number needed to treat and Relative risk reduction have been mentioned, which require correct interpretation in this context. 2) PLANNING: Now, let's start with understanding these terms: - Absolute risk reduction (ARR): It's the difference in the risk of an event happening between two groups (2.2% - 1.2% in this case). - Number needed to treat (NNT): It's the inverse of the absolute risk reduction (it tells us the number of patients that need to be treated to prevent one event). - Relative risk reduction (RRR): It's the proportion of the reduction of bad outcomes in the treated group compared to the control group in a clinical trial. With these definitions in mind, we know that we need to calculate the ARR, NNT, and RRR from the provided data and correlate them to the presented answer choices. 3) CLINICAL REASONING: Given the data, let's apply clinical reasoning. The incidence of myocardial infarction in the placebo group was 2.2% and in the aspirin group, it was 1.2%. This gives an absolute risk reduction (ARR) of (2.2% - 1.2%) = 1%. Now, the number needed to treat is (1 / ARR), which is (1 / 1%) = 100, so there isn't an accurate match in the options for this. The relative risk reduction (RRR) would be (ARR / rate in the control group) = (1% / 2.2%) = 0.4545 or about 45%, which again doesn't match correctly with the provided options. 4) CONCLUDING: Utilizing a step-by-step deductive clinical reasoning approach, we find that the most accurate statistical measure from the data is the Absolute Risk Reduction (ARR) which equals 1%. 5) RESPONSE: Therefore, from the given clinical scenario regarding the efficacy of aspirin in reducing myocardial infarction, we conclude that the most accurate interpretation of the results is (A) Absolute risk reduction is 1%.

**AI_E:** (1) RESTATING: This clinical trial observes the aspirin's efficacy in reducing the risk of myocardial infarction. It shows a 1.2% incidence in the group taking aspirin and a 2.2% incidence in the placebo group. In this situation, we need to interpret these results in terms of risk reduction or number needed to treat. Risk reduction is a measure of the decrease in risk of a specific disease in the exposed group (aspirin group in this case) compared to the unexposed group (placebo group). The number needed to treat (NNT) represents the estimated number of patients who need to be treated with a particular therapy for a specified period to prevent one additional adverse event or outcome. (2) PLANNING: Our primary assumption is that the lower incidence in the aspirin group does indeed signify a risk reduction due to the treatment. However, this needs to be quantified and interpreted in a manner that is clinically significant and accurate. To do this, we need to calculate the absolute risk reduction (ARR), relative risk reduction (RRR) and number needed to treat (NNT). Upon calculating these measures, we will be able to evaluate which interpretation fits the best among the given choices. (3) CLINICAL REASONING: The absolute risk reduction (ARR), sometimes called risk difference, is the absolute decrease in risk of a disease or unwanted condition in the treatment group compared to the control group. It is calculated as |CER - EER|, where CER is the control event rate and EER is the experimental event rate. In this case, it equals |2.2% - 1.2%| = 1%. The relative risk reduction (RRR) is the proportion of the risk reduction attributable to the intervention as compared to the control. It is calculated as (CER - EER) / CER. In this case, it equals (2.2% - 1.2%) / 2.2% = .45 or 45%. The number needed to treat (NNT) is the estimate of the number of patients that needs to be treated in order to have an impact on one person, calculated as 1 / ARR. Here, it equals 1 / 1% = 100. (4) CONCLUSION: According to the calculations, the Absolute Risk Reduction (ARR) is 1% which aligns with option A, the Relative Risk Reduction (RRR) is 45% which does not align with any of the given choices, and the Number Needed to Treat (NNT) is 100 which also does not align with any options. Therefore, the most accurate interpretation among the given choices would be option A. (5) RESPONSE: Given these estimates, the most accurate interpretation of these results is that the effective absolute risk reduction is 1% (Option A).

**Facilitator** The question asks for the most accurate interpretation of a clinical trial's results, which assessed the efficacy of aspirin in decreasing the risk for myocardial infarction among 22,000 male physicians with no history of coronary artery disease. The trial found that the incidence of myocardial infarction was 1.2% in the aspirin group and 2.2% in the placebo group. The reduction in incidence was statistically significant. To interpret these results, we need to calculate the Absolute Risk Reduction (ARR), Number Needed to Treat (NNT), and Relative Risk Reduction (RRR). The ARR is the difference in the risk of an event happening between two groups, which in this case is 2.2% - 1.2% = 1%. The NNT is the inverse of the ARR, which tells us the number of patients that need to be treated to prevent one event. In this case, the NNT is 1 / 1% = 100. The RRR is the proportion of the reduction of bad outcomes in the treated group compared to the control group in a clinical trial. Here, the RRR is 1% / 2.2% = 0.4545 or about 45%. Comparing these calculations with the provided answer choices, we find that the most accurate interpretation of the results is that the Absolute Risk Reduction (ARR) is 1% (Option A). The calculated NNT and RRR do not match any of the provided options. Therefore, the consensus statement is that the most accurate interpretation of these results is "Absolute risk reduction is 1%" (Option A).
